# Neuropsychiatric Comorbidity in Obesity: Role of Inflammatory Processes

**DOI:** 10.3389/fendo.2014.00074

**Published:** 2014-05-15

**Authors:** Nathalie Castanon, Julie Lasselin, Lucile Capuron

**Affiliations:** ^1^UMR 1286, Laboratory of Nutrition and Integrative Neurobiology (NutriNeuro), French National Institute for Agricultural Research (INRA), Bordeaux, France; ^2^UMR 1286, Laboratory of Nutrition and Integrative Neurobiology (NutriNeuro), University of Bordeaux, Bordeaux, France; ^3^Stress Research Institute (Stressforskningsinstitutet), Stockholm University, Stockholm, Sweden

**Keywords:** obesity, inflammation, neuroinflammation, cytokines, gut-brain axis, mood, cognition, neuropsychiatric symptoms

## Abstract

Neuropsychiatric symptoms are frequent in obesity. In addition to their substantial economic and health impact, these symptoms significantly interfere with the quality of life and social function of obese individuals. While the pathophysiological mechanisms underlying obesity-related neuropsychiatric symptoms are still under investigation and remain to be clearly identified, there is increasing evidence for a role of inflammatory processes. Obesity is characterized by a chronic low-grade inflammatory state that is likely to influence neuropsychiatric status given the well-known and highly documented effects of inflammation on brain activity/function and behavior. This hypothesis is supported by recent findings emanating from clinical investigations in obese subjects and from experimentations conducted in animal models of obesity. These studies converge to show that obesity-related inflammatory processes, originating either from the adipose tissue or gut microbiota environment, spread to the brain where they lead to substantial changes in neurocircuitry, neuroendocrine activity, neurotransmitter metabolism and activity, and neurogenesis. Together, these alterations contribute to shape the propitious bases for the development of obesity-related neuropsychiatric comorbidities.

## Introduction

The pandemic of obesity represents a major public health concern, as this disorder is associated with an increased risk of medical comorbidities contributing to a significant rise in mortality. Among those comorbidities related to obesity, neuropsychiatric disorders are particularly preoccupying. Not only neuropsychiatric symptoms affect the quality of life of obese subjects and contribute to their social impairment, but also they represent potent risk factors for aggravation of obesity. Given the growing prevalence of both obesity and neuropsychiatric disorders worldwide, the identification of the mechanisms underlying their comorbid association is urgently needed.

While different mechanisms are likely to be involved in the development of neuropsychiatric comorbidity in obesity, there is increasing evidence for a role of inflammatory processes. Chronic low-grade inflammation is an important characteristic of obesity and inflammatory processes are notorious for modulating brain functions and causing behavioral alterations. Recent clinical findings indicate that the increased systemic expression of inflammatory markers (e.g., cytokines) in obesity correlates with neuropsychiatric status, notably as it relates to mood and cognitive function. Moreover, studies in experimental animal models of obesity contribute to show that obesity-related inflammation manifests not only at the periphery but also within the brain where it modulates neurocircuitry, neurochemistry, and behavior. These findings that provide strong support to the notion that obesity-related inflammation plays an important role in the pathophysiology of neuropsychiatric symptoms will be presented and discussed in the present review.

## Neuropsychiatric Comorbidity in Obesity

Neuropsychiatric comorbidity, including mood and anxiety disorders, binge eating, and mild cognitive impairment, is frequent in obesity and is associated with a significant reduction in the quality of life and social functioning of obese individuals. Among those disorders often seen in obese subjects, depressive symptoms are particularly frequent with a prevalence rate significantly higher (up to 30%) compared to the general age-matched population (1–5). Similarly, reports of cognitive disturbances in obesity are multiple. Those alterations concern primarily planning function, problem solving, mental flexibility, and inhibitory processes, suggestive of frontal lobe alterations (6–9). Impairment in memory, regardless of age, has been also reported (10, 11). Associations between obesity and cognitive impairment have been more often reported in cross-sectional studies comparing performance from overweight/obese subjects to performance from lean participants. Nevertheless, a predictive longitudinal association of obesity with the development of age-related cognitive deficit has also been documented in several reports (12–14).

The directionality of the relationship between obesity and neuropsychiatric symptoms is usually difficult to determine from clinical studies. The significant improvement in mood and cognitive function reported after weight loss induced by bariatric surgery or diet restriction in obese subjects supports the hypothesis that obesity significantly impacts neuropsychiatric status and contributes to the development of neuropsychiatric comorbidity (15–22). Nevertheless, other reports indicate that preexisting mood and cognitive alterations can promote and/or predict the development of later obesity (23–26), attesting of the bidirectional link between obesity and neuropsychiatric status. To further address this issue, animal models of obesity represent certainly a useful and unique opportunity. Several models of obesity resulting either from genetic manipulations or diet modifications have been developed over the last decades (27). Among those models, diet-induced obesity (DIO) is probably the closest to human obesity with respect to etiological aspects. Moreover, because of its longitudinal characteristic, DIO allows the investigation of the mechanisms and pathophysiological changes preceding the development of obesity-related comorbidities, including neuropsychiatric alterations. Genetic models of obesity, in particular severe obesity, are also of great interest to explore the genetic–metabolic–brain interactions associated with obesity-related comorbidities. In that context, *ob*/*ob* (deficient for leptin) and *db*/*db* (deficient for functional leptin receptor) mice are particularly relevant as, in addition to metabolic disorders, these mice also display brain alterations (28–30). Overall, behavioral changes reported in experimental models of obesity include alterations in emotional reactivity and impairment in learning and memory [for review, see Ref. (31)]. Relevant to neural function, significant decreases in hippocampal-dependent learning together with impaired hippocampal neurogenesis and neuronal plasticity have been documented in animal models of DIO, notably in young mice (32–35). Similar results were described in *db*/*db* mice (28–30), supporting the notion that the hippocampus plays a major role in mediating obesity-associated cognitive impairment. Interestingly, *db*/*db* mice also display anxiety-like behavior (36). In contrast, depressive-like behavior appears to be mostly unchanged in animal models of obesity (36, 37), except in challenging conditions including stimulation of the immune system (38–40). Altogether, these data comfort the notion that neuropsychiatric comorbidities in obesity rely on interactions involving multiple systems, including metabolic characteristics, environmental influences, and immune-related processes.

## Obesity and Inflammation

Basal systemic low-grade inflammation is a fundamental characteristic of obesity, which is now considered not only as a metabolic disorder but also as an inflammatory condition affecting both the innate and acquired immune systems (41, 42). Obesity is characterized by increased levels of circulating proinflammatory cytokines [including interleukin (IL)-1β, tumor necrosis factor (TNF)-α, and IL-6], accumulation of leukocytes within the adipose tissue and other organs, activation of macrophages in the liver and fat, and activation of proinflammatory signaling pathways in multiple organs (43, 44). Inflammatory markers in obesity correlate more with measures of central adiposity, such as waist circumference and waist-to-hip ratio, rather than with the general measure of body mass index (BMI) (45–48). Interestingly, significant improvement in inflammatory profile is obtained after weight loss induced by low-caloric diet or bariatric surgery in obese individuals (49–54).

**Figure 1:**
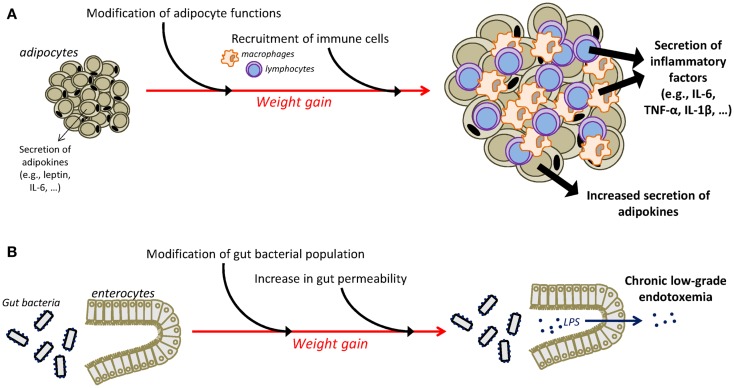
**Mechanisms underlying chronic low-grade inflammatory state in obesity**. The adipose tissue is an important contributor of chronic low-grade inflammation in obesity **(A)**. Weight gain is associated with substantial changes in the function of adipocytes that increase their secretion of adipokines, including inflammatory factors. Moreover, infiltration of immune cells, i.e., macrophages and T cells, in the adipose tissue is also responsible for the adipose secretion of inflammatory cytokines. Additional mechanisms, including alterations in the gut microbiota, contribute also to the instauration of obesity-related inflammation **(B)**. Obesity is associated with modifications in gut microbial population and with an increased permeability of the intestinal wall that promotes the passage of LPS in the circulation, leading thus to the development of chronic low-grade endotoxemia and the increased production of inflammatory factors. IL-1β, interleukin-1β; IL-6, interleukin-6; LPS, lipopolysaccharide; TNF-α, tumor necrosis factor-α.

Different mechanisms have been identified as playing a major role in the instauration of the chronic low-grade inflammatory state that characterizes obesity (Figure 1). One major player is the adipose tissue that has the ability to secrete adipokines and in which macrophages accumulate and potently secrete inflammatory factors (55–59). Moreover, an additional role for T cells in the development of adiposity-related inflammation is supported by several recent studies (57, 60–63). Obesity-related inflammation can also be triggered by pathogens, as there is now evidence of gut microbiota alterations associated with inflammatory processes in obesity (64, 65). In obese animals, gut microbial population is altered independently of diet characteristics (66), notably in the form of decreased *Bacteroidetes* and *Bifidobacterium* populations together with increases in the number of *Firmicutes* (64, 66, 67). High-fat diet is also associated with alterations in gut permeability leading to the instauration of a state of chronic low-grade endotoxemia (presence of lipopolysaccharide, LPS, in the blood) that is believed to contribute to obesity-related inflammation by the activation of systemic macrophages through the binding of LPS on TLR4 (68, 69). Similarly, recent clinical data in obese individuals indicate significant associations between gut microbiota modifications, reflected by the reduction in *Bacteroidetes/Firmicutes* ratio, and markers of local and systemic inflammation (67) and document improvement in the intestinal microbiota profile and in serum levels of an endotoxemia marker (LPS binding protein) following weight loss (70–72).

It is now clear that systemic inflammation in obesity contributes to both increased central inflammatory processes (notably in the hippocampus and hypothalamus) and metabolic dysregulations, including insulin-resistance. While these mechanisms are believed to play a major role in the development and maintenance of obesity (68, 73), their specific contribution to the development of the disorder is still under investigation. With respect to central inflammation, *db*/*db* mice that exhibit immune defects such as increased systemic inflammation and reduced immune competence (64, 74, 75) also show increased hippocampus cytokine expression (36, 37), and these effects are associated. Moreover, there is mounting evidence for enhanced cytokine expression and microglial activation in the hypothalamus in animal models of obesity (76–78). Consistent with these experimental findings, clinical indication of gliosis was recently reported in the mediobasal hypothalamus of obese individuals (79). While it is likely that central inflammation in obesity results from adiposity-related systemic inflammatory processes, the increases in circulating levels of TLR4 ligands and free saturated fatty acids following impaired gut permeability in high-fat diet mice (68, 80) may alternatively contribute to central inflammation, through activation of TLR4/MyD88 signaling (81, 82). Further investigation is needed to determine whether central activation of inflammatory processes occurs as a result of peripheral inflammation or whether it represents an early event promoting the development of obesity following a high-fat diet.

With regard to inflammation-related metabolic dysregulations, the inflammatory cytokine, TNF-α, was found to play an important role in the pathogenesis of insulin-resistance and type 2 diabetes (83–85). Inflammatory factors are also strong modulators of energy balance mainly due to their effects on the brain, in particular the hypothalamus (76), a mechanism that may promote weight gain through impairment of local peptidergic neuronal networks involved in food intake (73, 86). Interestingly, obesity-associated inflammation, notably as it relates to the visceral adipose tissue, was found to impact obesity treatment outcomes, with increased adipose expression of immune cells and inflammatory markers being associated with lower BMI reduction after bariatric surgery in severely obese patients (57).

## Central Effects of Inflammation: Evidence and Mechanisms

There is clear evidence for a role of proinflammatory cytokines in the development of neuropsychiatric symptoms (87). Proinflammatory cytokines released locally by activated innate immune cells have access to the brain, through different mechanisms that have been reviewed elsewhere (87). These pathways, which include humoral, neural, and cellular routes, ultimately lead to the production of cytokines by activated glial cells, in particular microglia, within the central nervous system (CNS). While microglia activation normally exerts a protective action on the CNS, its unregulated and chronic activation may in contrast become deleterious. Within the brain, proinflammatory cytokines activate the neuroendocrine system, impair neurotransmitter metabolism and function, and alter neural plasticity and brain circuitry (87). These biological alterations are associated with a large number of behavioral changes that have been referred to as sickness behavior (88). Necessary for the recovery of the host to the infection, sickness behavior usually resolves within few days. However, in cases of chronic and unregulated activation of the immune system, sickness behavior may evolve into clinically relevant neuropsychiatric symptoms, including depression and cognitive symptoms (88). At the clinical level, strong evidence for a role of cytokines in the development of neuropsychiatric comorbidity emanates from the model of cytokine therapy, based in particular on interferon (IFN)-α administration. Using this model, we and others have shown that IFN-α induces major depression in up to 45% of treated patients (89, 90) and that this effect relates on inflammation-induced alterations in the hypothalamic–pituitary–adrenal (HPA) axis, neurotransmitter function, and enzymatic pathways involved in the metabolism of monoamines (87, 91). Consistent with this last point, inflammatory factors are able to induce the synthesis of the enzymes indoleamine 2,3-dioxygenase (IDO) and GTP-cyclohydrolase 1 (GTP-CH1) in monocytes/macrophages and dendritic cells, which results in significant alterations in the biosynthesis of key monoamines (e.g., serotonin, dopamine) known to play a major role in mood regulation and cognitive function. Moreover, IDO is the first and rate-limiting enzyme that catabolizes tryptophan along the kynurenine pathway, a pathway leading ultimately to the production of neuroactive metabolites that have been associated with depressive symptoms in IFN-α-treated patients (92). In particular, IDO activation results in an increased production of the glutamatergic metabolites, 3-hydroxykynurenine, and quinolinic acid, which are well-known to induce neuronal death and to whom brain or cerebrospinal fluid concentrations were found to be increased in several neuropsychiatric or neurodegenerative diseases (93–96). Consistent with these data, inflammation-induced depressive- and anxiety-like behaviors in mice can be prevented by pharmacological or genetic inhibition of brain IDO activation (97–101). Moreover, NMDA receptor blockade abrogates cytokine-induced depressive-like behavior in mice (102). Interestingly, the hippocampus was found to play an important role in cytokine and IDO activation (103–106) and dysregulated activation of hippocampal microglia was associated with sustained IDO activity and protracted depressive-like behavior (104). Moreover, emotional alterations linked to inflammation-induced hippocampus IDO activation in mice was associated with reduced hippocampal expression of the brain-derived neurotrophic factor (BDNF) (107) that contributes to mood regulation and memory function. Altogether, these results point to a pivotal role of IDO activation, particularly in the hippocampus, in mediating cytokine-induced mood and cognitive alterations.

## Role of Inflammatory Processes in Obesity-Related Neuropsychiatric Symptoms: Clinical and Experimental Findings

Recent clinical findings support the hypothesis that inflammatory processes contribute to neuropsychiatric comorbidity in obesity, notably as it relates to mood status and cognitive function [see for review, Ref. (108)]. In support of this notion, concentrations of the inflammatory markers, C reactive protein (CRP) and IL-6, have been associated with depressive symptoms in obese subjects or in patients afflicted with the metabolic syndrome (109–111). Similar associations have been reported with leptin (112). Moreover, it was recently shown that CRP levels explained approximately 20% of the increase in depression scores over time in obese subjects (113). Consistent with the role of adiposity in these associations, reductions in inflammatory markers following weight loss induced by bariatric surgery were found to correlate with significant improvement in the emotional status and depression scores of severely obese individuals (114, 115). Given the bidirectional link reported between obesity and depressive symptoms, it is highly probable that depressive symptoms occurring in the context of obesity-related inflammation may in turn contribute to obesity maintenance, promoting thus the instauration of a vicious circle. Regarding cognitive function, a significant relationship was reported between CRP levels and decreased performance on cognitive tests targeting frontal lobe function in obese and overweight women (116). Moreover, in patients with the metabolic syndrome, higher levels of CRP and IL-6 were found to increase the risk of age-related cognitive decline (117). While these data support a role for obesity-related inflammation in the development of neuropsychiatric symptoms, the literature is still sparse regarding the causality of the events and the mechanisms that specifically underlie these effects in the context of obesity. Result from animal models of obesity may help to start to address this issue. In genetically or diet-induced obese rodents, increased cytokine expression in the hippocampus and cortex is associated with emotional and cognitive alterations (30, 31, 36, 37, 118). Interestingly, hippocampal IL-1β expression in *db*/*db* mice is related to adiposity and its blockade normalizes hippocampal dendritic spine density and prevents synaptic dysfunction and cognitive impairment (30). Associations have also been found between hippocampal microgliosis and obesity-related elevation in plasma glucocorticoids in the same mice (119). Consistent with the role of inflammatory processes, a direct relationship has been recently reported by our group between inflammation-related brain IDO activation and the development of depressive-like behavior in *db*/*db* mice (37). Similarly, we showed that DIO exacerbates both hippocampal induction of cytokines and IDO in response to an immune challenge and related behavioral changes (40). Interestingly, exacerbated depressive-like behavior is also associated in DIO mice with increased hypothalamic inflammation (39, 40). Beyond its impact on energy homeostasis, hypothalamic inflammation might also influence obesity-related emotional alterations. In addition to inflammatory processes, metabolic factors associated with obesity, including insulin or leptin, may also be able to act within the brain and lead to behavioral alterations (120). Nevertheless, several studies suggest that these factors *per se* are not sufficient to explain neuropsychiatric symptoms occurring in contexts of obesity. In support of this, increased emotional behaviors and cognitive impairment have been reported in animal models of obesity in the absence of any significant hyperinsulinemia (32, 36, 40). Reciprocally, the normalization of hyperglycemia in *db*/*db* mice was not effective in reversing spatial cognitive impairment or anxiety-like behavior (28, 29). Moreover, no difference in brain concentrations of glucose and insulin was measured in both *db*/*db* and *db/*+ mice and these concentrations remained the same when peripheral hyperinsulinemia was normalized (28). Altogether, these data point to brain inflammation as a major player in the development of obesity-related neuropsychiatric symptoms, although the pathways linking inflammation to these symptoms still need to be thoroughly studied.

## Inflammation-Driven Neuropsychiatric Comorbidity in Obesity: Potential Underlying Pathways

There are several pathways by which inflammation may promote the development of neuropsychiatric comorbidity in the context of obesity. Some of these mechanisms may be common to various situations of chronic inflammation and some may be more specific to the condition of obesity. Non-specific mechanisms include diffusion of inflammatory markers from the adipose tissue to the circulation and activation of relevant immune-to-brain pathways including humoral, neural, and cellular routes leading ultimately to the production *de novo* of inflammatory cytokines within the CNS and subsequent alterations in CNS functions (e.g., changes in neuroendocrine function, neurocircuitry, enzymatic pathways, and neurotransmitter metabolism/function) as described above. In particular, similar to other inflammatory conditions, obesity has been often associated with alterations in basal ganglia/reward circuitry and dopamine function (121, 122). Accordingly, several studies have indicated that obesity is associated with reduced striatal dopamine D2 receptor availability together with alterations in the fronto-striatal network (123–125). Moreover, investigations in rodents with DIO have shown significant associations between alterations in striatal circuitry and depressive-like behavior, suggesting a role for dopamine-related disruptions in obesity-associated depressive symptoms (38). Relevant to the contribution of inflammatory processes to these effects, the basal ganglia and dopamine system are highly targeted by inflammatory factors (126). In addition, inflammation-induced neuropsychiatric symptoms, in particular fatigue, anhedonia, psychomotor slowing, decreased motivation, and depressed mood, have been found to relate to alterations in basal ganglia/dopamine function and striatal circuitry in subjects treated with immune agents (127–129).

Alterations in neuroendocrine function – a mechanism highly described in the neurobiology of mood disorders – represent another common feature of inflammatory conditions, including obesity. In particular, obese subjects have been shown to exhibit an impaired feedback response to cortisol, similar to what is observed in depression (130). The immune system and neuroendocrine system are in constant communication and immune alterations are notorious for causing significant changes in neuroendocrine activity and *vice versa*. While the association between low-grade inflammation and alterations in the neuroendocrine system remains to be determined in obese subjects, it is highly possible that obesity-related neuroendocrine dysfunction contributes to neuropsychiatric comorbidity in obese individuals.

Converging findings have highlighted the consequences of deregulated hippocampal cytokines and neurotrophins expression on mood, learning, and memory (88, 131, 132), and the negative impact of cytokines on neurotrophins and synaptic plasticity (107, 133). Alterations in these mechanisms have been fully documented in models of chronic stress exposure, a well-admitted contributor of mood disorders (134, 135). Several clinical and experimental data strongly suggest that these deregulations may similarly participate to obesity-related neuropsychiatric alterations. Indeed, body weight loss induced by lifestyle intervention program in young obese patients normalizes plasma levels of BDNF (136). Moreover, cognitive impairment and emotional alterations reported in DIO and genetic models of obesity are linked to increased inflammation and reduced BDNF levels in the cortex (118) and the hippocampus (36, 37). Reciprocally, anti-inflammatory interventions in DIO mice reduce body weight, normalize hippocampal levels of BDNF, and prevent hippocampus-mediated cognitive impairments (137).

Alterations in the gut–brain axis represent one mechanism of inflammatory-driven neuropsychiatric comorbidity that may be more relevant to the condition of obesity. As already mentioned, obesity is associated with alterations in the gut microbiota in the form of modifications in microbiota populations, increased gut permeability, and activated inflammatory processes. A rich and complex communication network exists between the gut and the brain that involves endocrine, immune, and neural pathways (138), and there are now multiple evidences that impairment or dysregulation in the gut–brain axis impacts on mood and cognitive function (139). These data suggest that gut microbiota alterations found in obesity may modulate gut-to-brain communication pathways, leading thus to the development of neuropsychiatric comorbidity.

Altogether, these data are in favor of the involvement of inflammation-related complex non-exclusive pathophysiological processes in the development of neuropsychiatric symptoms in obesity. In that context, strategies to reduce inflammation either by pharmacological or non-pharmacological interventions (e.g., diet, surgical weight reduction strategies, exercise) may help in the prevention and management of obesity-related neuropsychiatric comorbidity. In particular, nutritional factors with immunomodulatory properties (i.e., omega-3 fatty acids, antioxidants) may worth being considered.

## Conclusion

**Figure 2:**
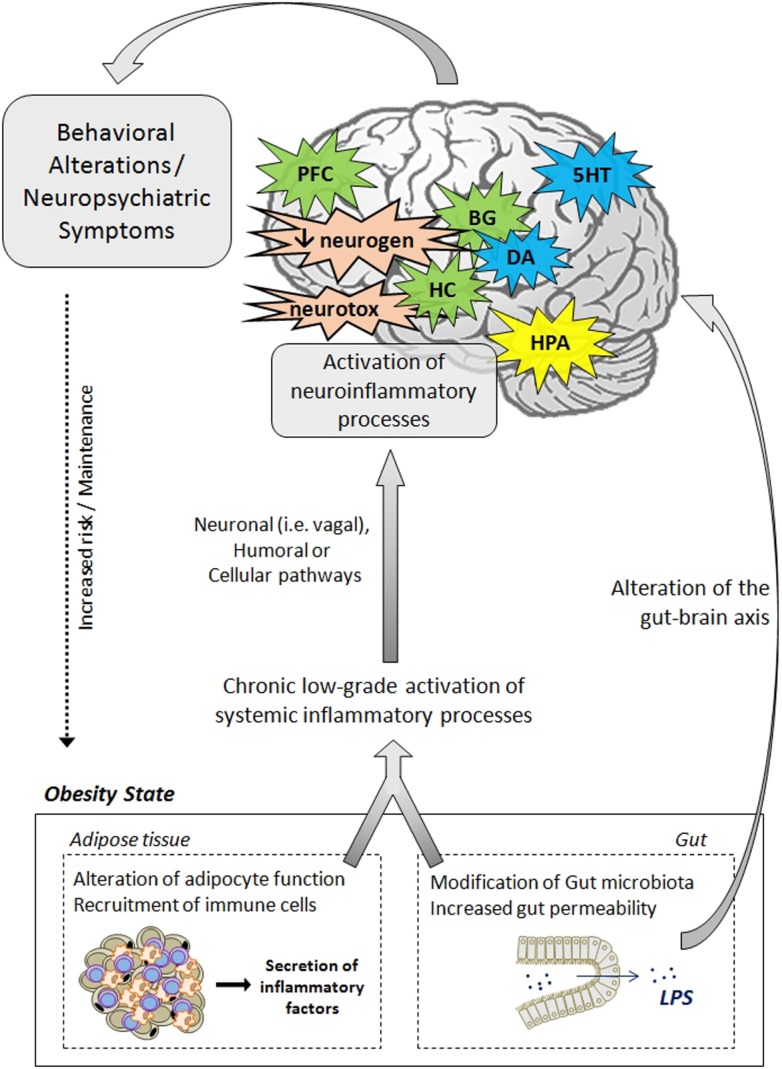
**Pathophysiological mechanisms likely to underlie neuropsychiatric comorbidities associated with obesity**. The activation of systemic inflammatory processes, originating from alterations in adipose tissue and gut functions, can contribute to the development of obesity-associated neuropsychiatric comorbidities. Proinflammatory cytokines released at the periphery can access the brain via several pathways (e.g., neural, humoral, and cellular routes) and induce the activation of neuroinflammatory processes, primarily by activating microglia. In the brain, proinflammatory cytokines impair neuroendocrine activity, neurotransmitter function (e.g., 5HT, DA, glutamate), and neurocircuitry, involving notably the hippocampus, the hypothalamus, the basal ganglia, and the prefrontal cortex. Cytokines can also disturb neurogenesis and induce neurotoxic effects through induction of IDO-derived neuroactive/neurotoxic metabolites. Altogether, these brain alterations lead ultimately to the development of behavioral/neuropsychiatric symptoms. Deregulations of the gut–brain axis, originating from changes in gut microbiota and permeability, may also contribute to mood and cognitive symptoms. These behavioral/neuropsychiatric symptoms can in turn promote the development or maintenance of obesity through risky or unadjusted eating behaviors. 5HT, serotonin; BG, basal ganglia; CNS, central nervous system; DA, dopamine; IDO, indoleamine 2,3-dioxygenase; HC, hippocampus; HPA, hypothalamic–pituitary–adrenal axis; LPS, lipopolysaccharide; PFC, prefrontal cortex; neurogen, neurogenesis; neurotox, neurotoxicity.

Data presented in this review strongly support the notion that inflammatory processes represent key players in the development of neuropsychiatric comorbidities in obesity. In addition to clinical investigations that clearly highlight the relationship between adiposity-related inflammation and neuropsychiatric symptoms in obese individuals, animal studies provide strong evidence of the direct effects of obesity-related neuroinflammatory processes on brain function and neurocircuitry and on the development of behavioral symptoms. The mechanisms and pathways leading to neuropsychiatric comorbidities in obesity are also discussed, starting from a general aspect to a viewpoint more specific to the condition of obesity. The effects rely on complex communication networks including the immune system, the gut, the neuroendocrine system, and key brain areas, including the hypothalamus, the hippocampus, and the basal ganglia (Figure 2). Alterations in monoamine metabolism and function, impaired neurotransmitter activity together with the occurrence of neurotoxic effects likely to promote neuronal death and decreased neurogenesis appear to represent major pathophysiological pathways to neuropsychiatric morbidity in obese individuals.

## Conflict of Interest Statement

The authors declare that the research was conducted in the absence of any commercial or financial relationships that could be construed as a potential conflict of interest.
